# Patients with psychosis spectrum disorders hospitalized during the COVID-19 pandemic unravel overlooked SARS-CoV-2 past infection clustering with HERV-W ENV expression and chronic inflammation

**DOI:** 10.1038/s41398-023-02575-3

**Published:** 2023-07-31

**Authors:** Ryad Tamouza, Urs Meyer, Alexandre Lucas, Jean Romain Richard, Irène Nkam, Armand Pinot, Ndilyam Djonouma, Wahid Boukouaci, Benjamin Charvet, Justine Pierquin, Joanna Brunel, Slim Fourati, Christophe Rodriguez, Caroline Barau, Philippe Le Corvoisier, Kawtar El Abdellati, Livia De Picker, Hervé Perron, Marion Leboyer

**Affiliations:** 1 AP-HP, Hôpital Henri Mondor, Département Médico-Universitaire de Psychiatrie et d’Addictologie (DMU IMPACT), Fédération Hospitalo-Universitaire de Médecine de Précision (FHU ADAPT), Créteil, F-94010 France; 2 Université Paris Est Créteil, INSERM U955, IMRB, Laboratoire Neuro-Psychiatrie translationnelle, F-94010 Créteil, France; 3 Fondation FondaMental, Créteil, France; 4 Institute of Pharmacology and Toxicology, University of Zurich-Vetsuisse, Zurich, Switzerland; 5 Neuroscience Center Zurich, University of Zurich and ETH Zurich, Zurich, Switzerland; 6 Institut des Maladies Métaboliques et Cardiovasculaires (I2MC), We-Met Platform, Inserm UMR1297 and Université Paul Sabatier, Toulouse, France; 7 GeNeuro, 18, chemin des Aulx, 1228 Plan-les-Ouates, Geneva, Switzerland; 8 Université de Lyon-UCBL, Lyon, France; 9 Virology Unit, Department of Prevention, Diagnosis and Treatment of Infections, Hôpital Henri Mondor (AP-HP) and Institut Mondor de Recherche Biomédicale, INSERM U955, Université Paris-Est, Créteil, France; 10 APHP, Hôpital Henri Mondor, Plateforme de Ressources Biologiques, F94010 Créteil, France; 11 Université Paris Est Créteil, Centre Investigation Clinique, CIC Henri Mondor, Créteil, F94010 France; 12 CAPRI, University of Antwerp, Antwerp, Belgium; 13 University Psychiatric Centre, Duffel, Belgium; 14 ECNP Immuno-NeuroPsychiatry Network, Utrecht, The Netherlands

**Keywords:** Predictive markers, Molecular neuroscience

## Abstract

Epidemiology has repeatedly associated certain infections with a risk of further developing psychiatric diseases. Such infections can activate retro-transposable genetic elements (HERV) known to trigger immune receptors and impair synaptic plasticity of neuroreceptors. Since the HERV-W ENV protein was recently shown to co-cluster with pro-inflammatory cytokines in a subgroup of patients with schizophrenia or bipolar disorder, we questioned the influence of the COVID-19 pandemic on patients with psychosis spectrum disorders (PSD). Present results revealed that (i) SARS-CoV-2 serology shows high prevalence and titers of antibodies in PSD, (ii) HERV-W ENV is detected in seropositive individuals only and (iii) SARS-CoV-2 and HERV-W ENV positivity co-clustered with high serum levels of pro-inflammatory cytokines in psychotic patients. These results thus suggest that SARS-CoV-2 infection in many patients with psychotic disorders now admitted in the psychiatry department did not cause severe COVID-19. They also confirm the previously reported association of elevated serum pro-inflammatory cytokines and HERV-W ENV in a subgroup of psychotic patients. In the context of the COVID-19 pandemic, this cluster is only found in SARS-CoV-2 seropositive PSD cases, suggesting a dominant influence of this virus on HERV-W ENV and cytokine expression, and/or patients’ greater susceptibility to SARS-CoV-2 infection. Further investigation on an interplay between this viral infection and the clinical evolution of such PSD patients is needed. However, this repeatedly defined subgroup of psychotic patients with a pro-inflammatory phenotype and HERV expression calls for a differential therapeutic approach in psychoses, therefore for further precision medicine development.

## Introduction

The coronavirus disease 2019 (COVID-19), caused by the Severe Acute Respiratory Syndrome CoronaVirus-2 (SARS-CoV-2), has generated unprecedented mortality and morbidity worldwide. It is now foreseen to probably become endemic as a multi-organ disease with a broad spectrum of manifestations [1], including long-term brain sequelae [2]. Similarly to previous post-coronavirus infection periods, survivors were described as having acute, but also persistent, neuropsychiatric (NP) impact of the infection [3] often unrelated to respiratory insufficiency and to COVID-19 severity [4]. The prevalence of psychosis during COVID-19 infection was estimated to be close to 0.9% in non-hospitalized patients and 2.8% for those admitted to intensive care units [5]. Among patients having recovered from COVID-19, a relatively large proportion (20–25%) is reported to suffer from long-lasting post-COVID symptoms, even after mild or asymptomatic forms of the disease. Post-COVID symptoms frequently include NP complications lasting months after the initial infection, strongly suggesting a persisting functional impairment of the brain [6]. The National Institute for Health and Care Excellence guidelines now defines post-COVID-19 syndrome as “signs and symptoms that develop during or after an infection consistent with COVID-19, persisting for more than 12 weeks (3 months) and not explained by an alternative diagnosis” [7]. Post-infection follow-up studies in Europe, USA or China reported that NP consequences were even more prevalent at, or beyond, 6 months (up to 34%) than after 3 months [8]. Meta-analysis showed that while symptoms such as anosmia, dysgeusia and headache resolve, new onset of sleep disturbances (30%), anxiety (23%) and depression (16%) are more frequent at 6 months or beyond and were predominantly observed in patients who had not been hospitalized for COVID [9]. Therefore, such long-term NP complications potentially reflecting brain neurobiological dysfunction several months after the initial infection, whatever the severity of the SARS-CoV-2 infection, require understanding the underlying causes to set up an early and accurate detection, along with a specific diagnosis allowing targeted treatments. These NP symptoms impact the quality of life and the social functioning of a significant proportion of the active population long after the pandemic era, now rather controlled by vaccination efforts but also feared to drift towards endemicity with an influenza-like scheme [1].

Different mechanisms by which SARS-CoV-2 can impact the brain have been described such as damaging endothelial cells leading to central nervous system thrombi and inflammation, which in turn can increase kynurenine, glutamate neurotoxicity, decrease monoamines and trophic factors, while activating complement cascade and microglia [2]. However, given that such dysregulations are likely neither attributable to a direct deleterious effect of a central SARS-CoV-2 infection nor to vascular and thrombotic events, it is of interest to uncover the underlying substratum of these neurobiological dysfunctions and NP symptoms.

Accordingly, it was recently shown that SARS-CoV-2 directly activates the expression of the Human Endogenous Retrovirus-W envelope protein (HERV-W ENV) in lymphoid cells on the one hand and that such a process was found in microglia of post-mortem brains from COVID-19 in the absence of any viral stigma [10]. HERV-W ENV was also found to be highly expressed in leukocytes of COVID-19 patients, with a magnitude paralleling that of inflammation, disease severity and outcomes [11]. These studies shed new light on the potential link between SARS-CoV-2 infection and post-COVID-19 outcomes.

HERV are genetic elements resulting from ancestral infections of germline cells, which represent 8% of the human genome. They are normally inactivated or epigenetically silenced, but some of their genes retain the ability to be activated. Nonetheless, several HERV sequences have been co-opted during evolution and play a physiological role, including an important contribution of defective copies to the non-coding regulatory RNAs. However, an abnormal expression of HERVs has been shown in various disorders including cancer, multiple sclerosis, diabetes, and rheumatoid arthritis. In particular, a HERV envelope protein, namely HERV-W ENV (W-ENV), was shown to be immunopathogenic and neurotoxic [12–14], the expression of which can be activated by certain exogenous viruses [15, 16]. W-ENV was shown to induce pro-inflammatory responses through the toll-like-receptor 4 cascade [17, 18] but also inhibition of oligodendrocyte precursor cell (re)myelination [19].

Thus, after having shown that W-ENV is detected in the blood of about one-half of patients with schizophrenia and one-third with bipolar disorder [20–22], we identified a W-ENV-induced pathway leading to glia- and cytokine-dependent major alteration of the N-methyl-d-aspartate receptor synaptic organization and plasticity [12]. This further showed that the W-ENV protein expressed in rat brains also induced abnormal behavioral patterns, which were inhibited/reversed and prevented by injections of a specific anti-HERV-W ENV antibody.

In the post-COVID-19 pandemic context, the present observational study aimed at exploring a possible relationship between exposure to SARS-CoV-2 and the expression of HERV-W ENV along with inflammation in a cohort of hospitalized patients suffering from psychosis without a known history of acute COVID infection. To this end, we quantified the circulating levels of W-ENV protein, of selected pro-inflammatory cytokines and determined the serological anti-SARS-CoV-2 IgG status in sera from patients having experienced a first episode or a relapse of a major mood or psychotic disorder during the early pandemic period, before the availability of anti-SARS-CoV-2 vaccine.

## Materials and methods

### Study cohorts

Forty-eight patients with psychosis spectrum disorder (PSD), with schizophrenia (*n* = 38) or with schizoaffective disorder (*n* = 10) were included in the present study while being hospitalized in the Department of Psychiatry of the Henri Mondor University Hospital (AP-HP, Créteil, France) for an acute psychotic episode, further referred to as PSD. As the serum collection was made during the first wave of COVID-19 in the spring of 2020 and before the vaccine against COVID was available, patients were confirmed to have a negative PCR test for SARS-CoV-2 when hospitalized. In addition, none of them could report a history of known COVID-19. The mean age of patients at inclusion was 41.67 ± 12.73 years, for the whole cohort (PSD), which comprised 35% females and 65% males. Psychiatric symptomatology was assessed using the French version the BPRS rating scale [23] by a trained psychologist or psychiatrist. Five milliliters of peripheral blood were drawn by venipuncture from subjects and allowed to clot for 1 h before centrifugation (1500 × g, 10 min). Samples were aliquoted from fresh serum and stored in at –80 °C and thawed on ice at the time of analysis.

A control group of 55 caregivers (CG, mainly nurses) working in the same hospital where patients were assessed and having been exposed to the same pandemic environment but without detectable COVID-19-related symptoms was included in the study. The mean age of CG at inclusion was 42.91 ± 10.76 years and 79.2% were females.

To avoid any interference either with the inflammatory status or with HERV expression, patients were enrolled based on the absence of any ongoing comorbid inflammatory or neurological disorder as well as the absence of any immunomodulatory treatment.

### Quantification of circulating levels of pro-inflammatory cytokines

Serum levels of cytokines were quantified with the fully automated immunoassay platform, Ella (ProteinSimple/Bio-Techne, CA, USA) with the Simple Plex Cytokine Storm Panel (Ref. # ST01A-PS-003229). The lower limits of quantification were provided by the supplier as a threshold for normal baselines, e.g., 3 pg/ml for IL-6. Serum samples were run in triplicate and results in pg/mL were calculated by the instrument software (Simple Plex Explorer, ProteinSimple/Bio-Techne, CA, USA).

### SARS-CoV-2 serology

The detection and quantification of anti-SARS-CoV-2 antibodies was performed by Simple Western technology, an automated capillary-based size electrophoretic sorting and immunolabeling system (Jess, ProteinSimple/Bio-Techne, CA, USA), with the SARS-CoV-2 Multi-Antigen Serology Module. All procedures were performed with the manufacturer’s reagents according to their recommendations. Briefly, a mix of his-tagged recombinant proteins of SARS-CoV-2 (Spike, S1 Subunit, S2 Subunit, S1RBD and Nucleocapsid) were used to detect specific anti-SARS-CoV-2 antibodies. Proteins were mixed with fluorescent master mix and heated at 95 °C for 5 min. Serums were diluted at 1/10 for IgG detection in serum diluent (ProteinSimple; SA-001). Anti-human IgG-HRP from ProteinSimple was used (ProteinSimple 043-491, ready to use). The denatured protein, blocking reagent, washing buffer, antibodies and chemiluminescent substrate were dispended into the microplate. Samples were loaded in duplicate into individual capillaries on a 25-capillary cartridge (12–230 kDa separation matrix). Protein separation and immunodetection were automatically performed on individual capillaries using default settings. The area under the curve (AUC) for the electropherograms peak corresponding to the immunodetection of each protein at the expected size, was measured using Compass Software. The threshold for positivity was equal to 1.2 × 107 total under the curve [AUC] values encompassing all SARS-CoV-2-related antigens, as determined by QC validation of the Kit.

### HERV-W ENV detection and quantification

For the detection of circulating serum HERV-W ENV antigen and the quantification of its circulating soluble form, all analyses were performed with the above-mentioned Simple Western system (Jess, ProteinSimple/Bio-Techne, CA, USA), according to the conditions provided in the patent published under ref. WO2019201908 (A1) and entitled “Method for the detection of the soluble hydrophilic oligomeric form of HERV-W envelope protein”, as previously described [24]. The specific signal was expressed as the signal-to-the-background-noise (S/N) ratio, where the background noise represents the mean + 2 SD of the background signals yielded by a panel of samples from healthy controls. According to the stability of the measured protein, the herein-studied serum samples were stored at –80 °C for less than 6 months. All samples were kept frozen after their initial freezing until use (first thawing cycle for the immunoassay).

### Statistical analyses

A detailed description of the statistical tests used is provided in the Supplementary Information. All statistical analyses were performed using SPSS Statistics (version 25.0, IBM, Armonk, NY, USA) and Prism (version 8.0; GraphPad Software, La Jolla, California), with statistical significance set at *p* < 0.05.

Of note, when some sera could not be tested because of quantity limitation or technical issues for test interpretation with requested QC criteria, the numbers analyzed in the successive analyses may differ from one to two PSD or CG.

## Results

**Fig. 1:**
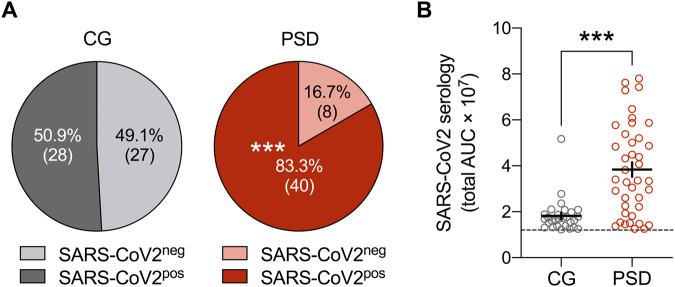
Distribution and characteristics of SARS-CoV-2 serology in caregivers (CG) and patients with psychosis spectrum disorder (PSD). **A** Percentage of CG and PSD subjects who were found to be negative (SARS-CoV-2^neg^) or positive (SARS-CoV-2^pos^) for SARS-CoV-2 based on SARS-CoV-2 IgG serology (threshold for positivity: >1.2 × 10^7^ total under the curve [AUC] values encompassing all SARS-CoV-2-related antigens). The numbers in brackets represent the number of subjects in each subgroup. ***p < 0.01, reflecting the significant difference in the number of SARS-CoV-2^pos^ cases in the PSD compared to the CG group, based on yate’s corrected *χ*^2^ test. **B** Cumulated SARS-CoV-2 serology values (total AUC values) in SARS-CoV-2^pos^ CG (*n* = 28) and PSD (*n* = 40) subjects. The dashed line represents the threshold for SARS-CoV-2 positivity. ***p < 0.001, based on the Mann–Whitney test.

Although no COVID-19 infection was recorded in patients and caregivers, we observed a positive anti-SARS-CoV-2 serology in the two studied subgroups (Fig. 1), indicating a previous infection or a significant response after exposure to SARS-CoV-2. Patients with psychotic disorder (PSD) had a significantly higher number of positive sera than caregivers (40/48, 83.33% and 28/55 50.90% respectively; *χ*^2^ with Yates correction: 10.6098. *p* = 0.001125; Fig. 1A) along a higher titer of specific anti-SARS-CoV-2 antibodies (Mann–Whitney *U* test, *p* < 0.001; Fig. 1B).

**Fig. 2:**
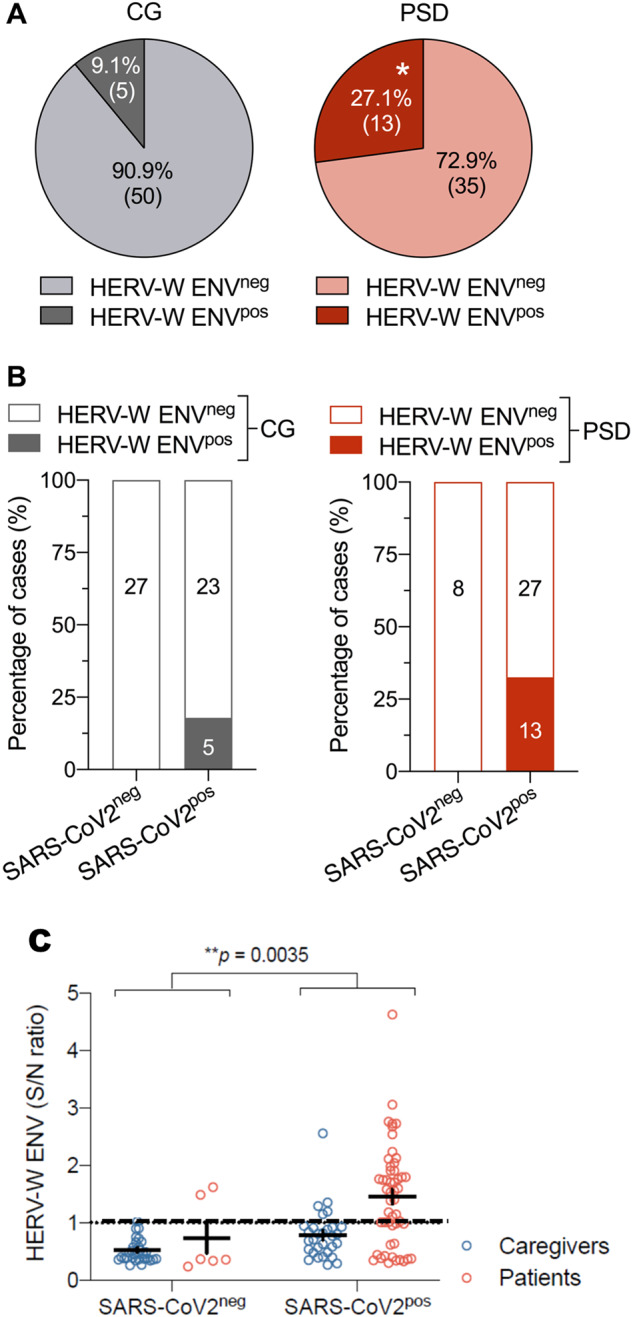
Distribution of HERV-W ENV protein in caregivers (CG) and patients with psychosis spectrum disorder (PSD). **A** Percentage of CG and PSD subjects who were negative (HERV-W ENV^neg^) or positive (HERV-W ENV^pos^) for HERV-W ENV protein in serum. The embedded numbers reflect the number of subjects in each subgroup. *p < 0.05, reflecting the significant difference in the number of HERV-W ENV^pos^ cases in the PSD compared to the CG group, based on the Mann–Whitney test. **B** Percentage of HERV-W ENV^neg^ and HERV-W ENV^pos^ cases in the CG and PSD groups split by SARS-CoV-2 positivity. The embedded numbers reflect the number of subjects in each subgroup. SARS-CoV-2 positivity was based on SARS-CoV-2 IgG serology (threshold for positivity: >1.2 × 10^7^ total area under the curve [AUC] values encompassing all SARS-CoV-2-related antigens). **C** HERV-W signal-to-noise ratio (S/N) comparing patients and caregivers within SARS-CoV-2-negative and -positive subgroups. **p = 0.0035, reflecting the significant difference in the number of HERV-W ENV^pos^ cases in comparing all seropositive versus all seronegative individuals for SARS-CoV-2, based on the Mann–Whitney test.

We further analyzed the same sera for the presence of HERV-W ENV protein (W-ENV) and found that a significantly increased proportion of SARS-CoV-2 seropositive PSD patients was W-ENV positive (13/48; 27.1%) as compared to CG (5/55, 9%). Of note, the SARS-CoV-2 seronegative CG had no detectable circulating W-ENV (0/27). PSD patients presented significantly elevated W-ENV levels as compared to the whole control group (Mann–Whitney *U* test, *p* = 0.0035; Fig. 2C). Among control caregivers who have positive SARS-CoV-2 serology, only low HERV-W ENV levels were found in four seropositive caregivers (9%; 5/55) with a single outlier (Fig. 2B).

**Fig. 3:**
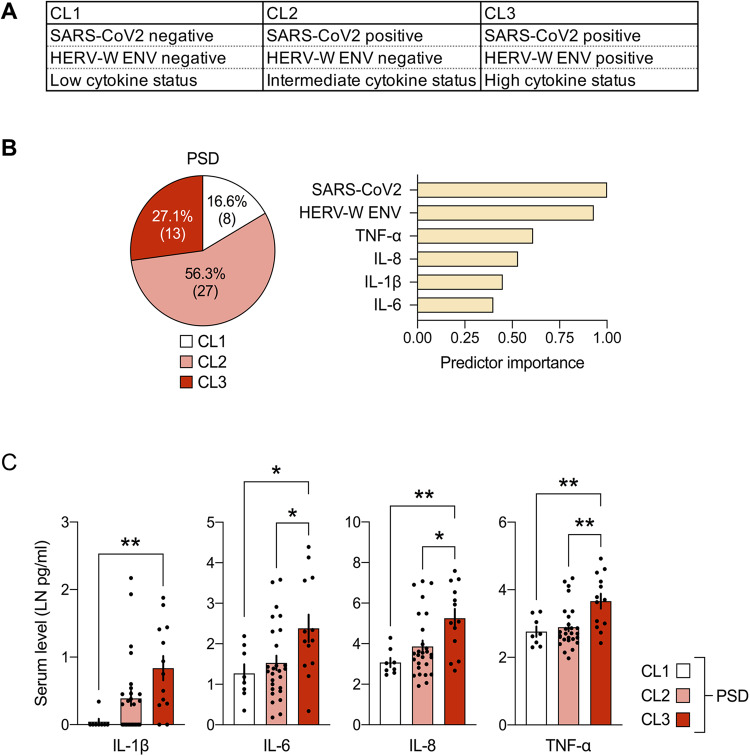
Stratification of patients with psychosis spectrum disorder (PSD) using unsupervised two-step cluster analysis. SARS-CoV-2 positivity (threshold for positivity: >1.2 × 10^7^ total under the curve values encompassing all SARS-CoV-2-related antigens), HERV-W ENV positivity (signal-to-noise [S/N] ratio >1) and serum cytokines (LN-transformed IL-1β, IL-6, IL-8 and TNF-α) were used as clustering variables. **A** Summary of the cluster characteristics defining CL1, CL2 and CL3. **B** The pie chart shows the cluster distribution of PSD subjects (*n* = 48) across the three clusters (CL1, CL2 and CL3) identified by two-step cluster analysis. The numbers in brackets represent the number of PSD subjects in each cluster. The bar plot shows the relative predictor importance for cluster separation as revealed by two-step cluster analysis. **C** Serum cytokine levels (LN-transformed; means ± SEM with individual values overlaid) in the three clusters (CL1, CL2, and CL3) of PSD patients. *p < 0.05 and **p < 0.01, based on Tukey’s post-hoc tests for multiple comparisons following one-way ANOVA.

To analyze the potential impact of SARS-CoV-2 seropositivity on serum circulating levels of W-ENV in association with pro-inflammatory cytokines, we performed cluster analyses of PSD patients as previously described in patients with psychosis before the COVID-19 pandemic [22]. The stratification of patients was performed using unsupervised two-step cluster analysis with the following clustering variables: SARS-CoV-2 seropositivity, HERV-W ENV positivity and pro-inflammatory cytokines levels (IL-1β, IL-6, IL-8 and TNF-α) as presented in Fig. 3. The analysis involved 48 PSD patients and identified three clusters, namely cluster 1 (CL1), CL2 and CL3 (Fig. 3A). The cluster 1 (16.6%; 8/48) corresponds to patients negative for both SARS-CoV-2 antibodies and HERV-W ENV antigenemia, along with low serum levels of pro-inflammatory cytokines. Cluster 2 (56.3%; 27/48) consists of patients with positive SARS-CoV-2 serology but negative HERV-W ENV antigenemia, and low/intermediate values of pro-inflammatory cytokines. Cluster 3 (27.1%; 13/48) represents patients positive for the three clustering variables with higher levels as compared to the two other clusters. We also observed that SARS-CoV-2 serology and HERV-W ENV protein had a dominant predictor importance for cluster identification followed by two cytokines, i.e., TNF-α and IL-8 (Fig. 3B).

This clustered distribution of patients thus showed three phenotypes significantly and consistently stratified on all three variables. Among cytokines, TNF-α distribution best fitted that of SARS-CoV-2 antibodies and HERV-W ENV protein levels with highly significant differences between CL3 and either CL1 or CL2 (Tukey’s post-hoc tests for multiple comparisons following one-way ANOVA, *p* < 0.01; Fig. 3C). Nonetheless IL-8 and Il-6 levels were also significantly elevated in CL3, compared to CL1 and CL2 (Tukey’s post-hoc tests for multiple comparisons following one-way ANOVA, *p* < 0.01 and *p* < 0.05, respectively). Interestingly, Il-1 beta provided a significant difference between CL3 and CL1 (Tukey’s post-hoc tests for multiple comparisons following one-way ANOVA, *p* < 0.01) while being negative in all CG sera but one with a low level (Fig. 3C).

## Discussion

A long-lasting history of NP syndromes or symptoms following viral pandemics has already been reported such as, for example, the “Spanish flu” [25, 26] during the last century, or the H1N1 pandemic [27]. More recently, mounting evidence showed that patients with psychotic disorders represent a particularly exposed population group to the SARS-CoV-2 infection, more at risk of being infected, which exhibited a higher rate of morbidity and mortality during COVID-19 episodes, particularly during the first pandemic waves [28].

The present study further provides compelling results showing that most psychotic patients with an a priori negative history of SARS-CoV-2 infection and not yet vaccinated against SARS-CoV-2 had high levels of anti-SARS-CoV-2 antibodies, which was not seen in caregivers similarly exposed to the same pandemic pressure. The observed presence of significantly elevated titers of anti-SARS-CoV-2 antibodies against specific and multiple antigens in the panel used for the serological analysis also validated its specific threshold (Cf. Mat. and Meth.) and ruled out the possibility of previous exposition to other strains of coronavirus with antibody cross-reactivity. One may therefore question the clinical expression of COVID-19 in these patients, which may either present a true psychiatric expression of the SARS-CoV-2 infection or a silent/mild clinical expression known to represent an important proportion of cases in certain conditions or areas [29]. To support such assumptions, the multiple studies showing that post-COVID disorders are not proportionate in incidence and in magnitude with the presence or absence of symptoms during acute COVID-19 infection are of particular relevance [30–32]. Concerning HERV-W activation, though it was found to correlate the severity of the first COVID-19 episode with all successive variants and to represent 100% of most severe cases, this activation along with the detection of the HERV-W ENV protein in serum or on blood lymphocytes was nonetheless detected in a significant percentage of patients with milder COVID-19 [10, 11, 33]. This is why the possibility that patients being treated with certain antipsychotic drugs before the infection with SARS-CoV-2 may have benefitted from their known antiviral properties, should be taken into account [34–38]. These antipsychotic drugs may have masked or lessened the symptomatology related to the infection per se, which would explain why no severe or significantly symptomatic COVID-19 cases were reported among the HERV-W ENV-positive psychiatric patients in this cohort. In addition, psychiatric patients are prone to pass health systems filters given the well-known stigmatization of patients with mental disorders and/or their isolation [39].

We here observed that the SARS-CoV-2-positive patients’ group exhibited higher circulating HERV-W ENV levels when compared to the caregiver seropositive group and to the SARS-Cov-2-negative psychotic patients. It is now well admitted that psychotic disorders, at least for substantial subsets of patients, are underpinned by immune dysregulations likely resulting from complex interactions between inter-individual genetic susceptibility and environmental stressors with ensuing pro-inflammatory processes [40]. From the results of previous studies [12, 20–22] and adding the present one, it is thus conceivable that the HERV elements may be at the crossroad of such interactions. A previous study showed that high IL-6 serum levels are correlated with HERV-W ENV antigenemia in COVID-19 and, in vitro, that IL-6 can be induced by SARS-CoV-2 in healthy donor’s PBMC but it appeared much later that HERV activation in “responding” donors [10]. Thus, in infected patients, the pro-inflammatory effect of HERV-W ENV protein is most probably superadding to the response to a viral infection and is likely to emphasize this cytokine expression beyond the normal range of immune responses. From this post-COVID pandemic period, we hence established a strong correlation in psychotic patients, between SARS-CoV-2 infection and HERV-ENV expression, along with elevated cytokine serum levels persisting in the absence of ongoing infection.

The present observation enriches the previously shown association between HERV expression and infectious triggers mostly consisting of common pathogens and including *Herpesviridae* strains such as HHV6, HSV1 and Epstein–Barr virus (EBV) [15, 16, 41] or Toxoplasma parasites [42]. Moreover, adding to the demonstration of an epidemiological association of EBV with the development of multiple sclerosis (Science, 2022 [43]), a recent massive screening of medical records established strong links between common viral infections and the development of neurological diseases including, Parkinson, Alzheimer, or multiple sclerosis [44]. Although this study leaves open the mechanistic pathway, it is worth reminding that HERV-W reactivation is one of the operative processes underlying the development of multiple sclerosis [13, 19], which was also shown to be activated by EBV membrane glycoprotein [16]. The present observations could hence be discussed according to the already shown involvement of HERV elements in psychiatric disorders and/or to the recently demonstrated ability of the SARS-CoV-2 to induce HERV-ENV expression by blood lymphoid cells [10, 11]. In this context, we have repeatedly shown that both SZ and BD disorders are underpinned by HERV-W-mediated processes [20, 21] with the recent identification of a signature consisting of HERV-W positivity, elevated level of circulating pro-inflammatory cytokines and history of childhood maltreatment [22]. Of note, as childhood maltreatment events are well known to be associated with persisting inflammation a long time after the triggering event [45], it is therefore possible that the presently observed inflammation may result from both HERV and childhood trauma in a yet to be determined sequential manner. Similarly, the presently observed increased level of HERV-W ENV in psychosis may still be due to the enhancement of a pre-existing HERV-W positive expression under the influence of SARS-CoV-2.

Possibly reflecting this question of their relative contributions, the analyzed cytokines found to discriminate HERV-W positive and negative groups of “pre-pandemic” patients with SZ or BD, had shown a strong association for IL-1 beta in BD or IL-6 in SZ. In the present study, the observed major driving effect of TNF-α and IL-8 could reflect inflammation mediated by SARS-CoV-2 infection per se as a pivotal marker of the cytokine storm characterizing COVID-19 infection. However, the lower relevance of IL-6, while IL-8 was shown to be a reliable maker of its mild forms [46], does not fit with an acute COVID-19 infection, even asymptomatic or masked by the treatments. Of potential relevance, IL-8, IL-6 and IL-1 were shown to reflect HERV-W expression in endothelial cells [17, 47].

However, limitations of the present study must be acknowledged: (i) the temporal sequence of HERV-W ENV, pro-inflammatory cytokines and SARS-CoV-2 interplay should be studied from a longitudinal study, (ii) the absence of information concerning a previous HERV-W ENV status and the potentially pre-existing inflammatory profile that characterizes subgroups of patients with psychosis, (iii) a non-recorded or silent COVID-19 infection just before hospitalization of patients in the psychiatric department and (iv) the present findings exclusively result from peripheral blood evaluation and, as far as possible and feasible, such a study could be improved by more direct analyses of neuro-inflammation including CSF and/or brain imaging analyses.

Whatsoever, a major translational perspective arises from this study by measuring the presently HERV-associated panel of biomarkers in post-COVID-19 patients with NP disorders, which could be translated into concrete medical diagnostic and then into therapeutic indications.

In conclusion, further characterization with the present variables, SARS-CoV-2 serology, HERV-W ENV and serum cytokines quantification may altogether allow a useful identification of consistent clusters, like in the present study, for optimizing a precision medicine strategy addressing a heterogeneous population of patients and, particularly, those with post-COVID-19 NP symptoms.

## Supplementary information

Supplementary material
