# Supplementary material for: Patients with psychosis spectrum disorders hospitalized during the COVID-19 pandemic unravel overlooked SARS-CoV-2 past infection clustering with HERV-W ENV expression and chronic inflammation
Source: Transl Psychiatry. 2023 Jul 31;13:272. doi: 10.1038/s41398-023-02575-3 (PMC10390536; doi:10.1038/s41398-023-02575-3)
Supplement: Supplementary file 1 — Supplementary material [file 41398_2023_2575_MOESM1_ESM.docx]

**Supplementary Information**

***Statistical analyses***

Demographic characteristics of study participants and HERV-W antigenemia were analyzed by chi-square tests. Two-step cluster analysis was used to identify possible subgroups of patients and controls with differing HERV-W positivity, serum cytokines and/or SARS-CoV-2 serology. The two‐step cluster method uses a pre‐clustering step by scanning the entire dataset and storing the dense regions of data records in terms of summary statistics, after which a hierarchical clustering algorithm is then applied to the cluster dense regions (1,2). Hence, it represents a hybrid approach, which first uses a distance measure to separate groups and then a probabilistic approach to choose the optimal subgroup model. The two-step cluster method was chosen for two main reasons. First, it is capable of handling data sets that are composed of a mixture of categorical (e.g. HERV-W positivity) and continuous (e.g. serum cytokines and SARS-CoV-2 serology) variables (1,2). Second, it can be run without predetermining the number of clusters, thereby avoiding bias in terms of identifying the number of possible clusters (Kent et al., 2014; Roy et al., 2007). For data clustering, we concomitantly integrated measures of HERV-W positivity (CO: IESR > 15; categorical variable), serum cytokines (IL-1β, IL-6, IL-8 and TNF-α, continuous variables) and SARS-CoV-2 serology (anti-Nucleocapsid and anti-Spike IgG levels; continuous variables) from PSD and CG subjects. Serum cytokines were first subjected to natural logarithmic transformation (LN) to minimize data skewness. Bayesian Criterion (BIC) was used to estimate of the maximum number of clusters, whereas the log­likelihood method was used as distance measure (Kent et al., 2014; Purves-Tyson et al., 2021; Roy et al., 2007).

Gelbard, R., Goldman, O., and Spiegler, I. (2007). Investigating diversity of clustering methods: an empirical comparison. Data Knowl. Eng. 63, 155–166. doi: 10.1016/j.datak.2007.01.002

Kent, P., Jensen, R. K., and Kongsted, A. (2014). A comparison of three clustering methods for finding subgroups in MRI, SMS or clinical data: SPSS twostep cluster analysis, latent Gold and SNOB. BMC Med. Res. Methodol. 14:113. doi: 10.1186/1471-2288-14-113

1. Purves-Tyson TD, Weber-Stadlbauer U, Richetto J, et al. Increased levels of midbrain immune-related transcripts in schizophrenia and in murine offspring after maternal immune activation [published online ahead of print, 2019 Jun 5]. Mol Psychiatry. 2019;10.1038/s41380-019-0434-0.

Kent, P., R.K. Jensen, and A. Kongsted. 2014. A comparison of three clustering methods for finding subgroups in MRI, SMS or clinical data: SPSS TwoStep Cluster analysis, Latent Gold and SNOB. *BMC Med Res Methodol* 14:113.

Purves-Tyson, T.D., U. Weber-Stadlbauer, J. Richetto, D.A. Rothmond, M.A. Labouesse, M. Polesel, K. Robinson, C. Shannon Weickert, and U. Meyer. 2021. Increased levels of midbrain immune-related transcripts in schizophrenia and in murine offspring after maternal immune activation. *Mol Psychiatry* 26:849-863.

Roy, G., G. Orit, and S. Israel. 2007. Investigating diversity of clustering methods: An empirical comparison. *Data & Knowledge Engineering* 63:155-166.
